# Flexural behavior and crack development of reinforced geopolymer slabs with longitudinal voids: an experimental study

**DOI:** 10.1038/s41598-026-53647-5

**Published:** 2026-05-24

**Authors:** Yasmin Hefni Abdel Aziz, Abeer El Malky, Taha A. El-Sayed

**Affiliations:** 1 Civil Engineering Department, Faculty of Engineering, Modern University for Technology & Information, Cairo , Egypt; 2 Civil Engineering Department, Shoubra Faculty of Engineering, Benha University, Banha, Egypt

**Keywords:** Geopolymer concrete, Hollow-core slabs, Flexural behavior, Cracking load, Void ratio, Sustainability, Engineering, Materials science

## Abstract

Geopolymer concrete, produced from the alkali activation of aluminosilicate-rich by-products such as fly ash, presents a sustainable, low-carbon substitute for standard Portland cement. Despite its acknowledged mechanical and durability advantages, researchers have not thoroughly examined the flexural performance of reinforced GC slabs particularly hollow-core designs. This paper offers a comprehensive experimental and theoretical assessment of reinforced GC solid and hollow-core slabs to fill this gap. Seven slabs were tested under four-point loading tests: two solid specimens (OPC and GC) and five hollow-core GC slabs featuring varying void sizes and shear-span-to-depth ratios (a/d). The investigation concentrated on reaction to cracking, ultimate flexural capacity, stiffness characteristics, and deflection behavior, facilitating a systematic evaluation of both material and geometric effects. The results indicate that the GC solid slab exhibited marginally superior flexural performance compared to the OPC slab, demonstrating advantageous bond properties and material uniformity. In hollow-core slabs, elevated void ratios resulted in significant decreases in cracking load, ultimate capacity, and effective stiffness, whereas alterations in a/d caused pronounced differences in strength and deflection characteristics. Reduced shear spans improved load capacity and stiffness, while extended spans led to a more pliable load–deflection response. Analytical predictions derived from traditional flexural theory closely aligned with experimental outcomes, validating the appropriateness of classical models for GC and voided slab systems. The results show that geopolymer concrete is a structurally sound and environmentally friendly alternative to regular Portland cement (OPC). Its efficacy in both solid and hollow-core configurations facilitate its wider implementation in contemporary low-carbon structural applications.

## Introduction

Hollow-core slabs (HCS) are precast concrete structural elements characterized by continuous longitudinal voids extending along the slab length, which significantly reduce self-weight while maintaining adequate structural depth and load-bearing capacity. The structural efficiency of HCS is achieved through solid webs between the voids, enabling effective stress transfer under bending actions. Acting primarily as one-way spanning members, hollow-core slabs exhibit a high stiffness-to-weight ratio and efficient material utilization, making them particularly suitable for long-span floor systems without intermediate supports. Experimental and analytical investigations have demonstrated that optimized HCS geometry can provide flexural capacities comparable to solid slabs while achieving substantial reductions in dead load^1–4^.

Reinforced concrete slab systems incorporating voids—such as bubble-deck, waffle, and hollow-core slabs—have been extensively studied as effective solutions for reducing structural self-weight while maintaining satisfactory flexural performance. Among these systems, HCS are widely adopted due to their construction efficiency, flexibility in accommodating electrical and mechanical services, and cost-effectiveness in multi-story buildings. The precasting process offers advantages such as improved quality control, dimensional accuracy, and rapid construction. Traditionally, hollow-core slabs are manufactured using Ordinary Portland Cement (OPC), which has demonstrated reliable structural performance but contributes significantly to global carbon emissions^5,6^. Consequently, increasing emphasis has been placed on developing sustainable alternatives for precast floor systems.

The flexural behavior of hollow-core slabs is strongly influenced by the geometry and configuration of internal voids. Under flexural loading, cracking typically initiates in the tensile zone and propagates toward the compression zone, following classical reinforced concrete behavior. However, the presence of voids modifies internal stress distribution, affecting stiffness, crack spacing, crack width, and ultimate load-carrying capacity. Previous experimental and numerical studies have shown that increasing void size improves weight efficiency but may reduce flexural stiffness and strength if not properly optimized^1,2,7,8^. Well-designed void configurations can achieve an effective balance between structural performance and material efficiency, resulting in improved crack control and reduced mid-span deflections.

In precast hollow-core slab systems, overall structural performance is governed not only by slab geometry but also by the efficiency of connections and composite action. Connections between adjacent slab units, supporting beams, and any structural topping layers play a critical role in load transfer, stiffness enhancement, and crack control. Proper connection detailing ensures structural continuity and enhances composite action, leading to delayed crack initiation and improved flexural response. In contrast, inadequate connection design may result in premature cracking, excessive deflections, and reduced load-carrying capacity. Design guidelines and previous studies emphasize that efficient connection systems are essential for ensuring reliable performance of hollow-core slabs in practical applications^4,6,9^.

Despite extensive research on conventional OPC-based hollow-core slabs, investigations focusing on geopolymer-based hollow-core slab systems remain limited. Existing studies predominantly address prestressed concrete HCS or geopolymer solid structural members, while experimental research evaluating flexural behavior, crack development, and connection efficiency in geopolymer hollow-core slabs is scarce^2,5,7^. Moreover, detailed crack pattern analysis and systematic assessment of composite action in geopolymer HCS are largely absent from the literature. This research gap highlights the need for comprehensive experimental studies to evaluate geopolymer hollow-core slabs as sustainable precast floor systems capable of achieving structural performance comparable to conventional solutions.

Geopolymer concrete is a cementless binder produced through the alkaline activation of aluminosilicate-rich materials. Various natural and industrial by-products have been utilized as precursors, including fly ash (FA)^10,11^, ground granulated blast furnace slag^12^, metakaolin^13^, red clay^14^, and mining residues^15^. Among these materials, fly ash is the most commonly used precursor due to its availability, low cost, and high reactivity^10,11^. The incorporation of FA in GC significantly reduces carbon emissions associated with cement production, promotes the reuse of industrial by-products, and mitigates disposal challenges related to FA waste^16^. The high silica and alumina content of FA accelerates geopolymerization, resulting in binders with improved mechanical, physical, and durability properties.

In addition to its environmental advantages, FA-based GC has demonstrated satisfactory mechanical performance. Several studies have reported high compressive strength, along with superior resistance to chemical attack and fire exposure. Pavithra et al.^17^ reported a compressive strength of 54 MPa for FA-based GC after 28 days. Hassan et al.^18^ observed that the elastic modulus of GC correlates well with compressive strength and is comparable to that of OPC concrete. Similarly, Verma et al.^19^ concluded that although GC may exhibit a slightly lower elastic modulus, its residual mechanical properties remain acceptable for structural applications.

Despite these advantages, FA-based GC generally exhibits reduced tensile strength and fracture toughness, leading to relatively brittle failure behavior^20^. To address this limitation, various fibers—including steel, polyvinyl alcohol, and natural fibers—have been incorporated into GC to enhance its ductility. In recent studies, waste rubber has also been introduced into GC to improve sustainability and address the environmental challenges associated with tire disposal^20^. However, the inclusion of rubber particles often results in reduced mechanical strength due to poor interfacial bonding between the rubber and geopolymer matrix^21^. Jokar et al.^22^ demonstrated that treating rubber particles with a 1 M NaOH solution significantly improves adhesion and mechanical performance.

Although extensive research has been conducted on geopolymer concrete beams, columns, and walls, limited studies have addressed the structural performance of steel-reinforced geopolymer concrete slabs, particularly precast hollow-core systems. Moreover, comprehensive comparisons between reinforced geopolymer concrete and conventional OPC concrete slabs remain scarce. Previous experimental and numerical studies on HCS have shown that void configuration—such as shape, size, and distribution—significantly influences flexural behavior, stiffness-to-weight ratio, and mid-span deflection^2,7^. Existing research also indicates that geopolymer concrete members can achieve structural performance comparable to OPC concrete when properly designed and reinforced^5,9,23^.

The present study incorporates treated rubber fibers derived from waste tires into the geopolymer concrete matrix. Rubber fibers have gained attention in sustainable concrete research due to two primary benefits: (1) diverting end-of-life tires from landfills or incineration, and (2) enhancing the ductility and post-cracking behavior of otherwise brittle geopolymer matrices^21,22^. However, untreated rubber typically exhibits poor interfacial bonding with cementitious binders due to its hydrophobic nature, leading to substantial reductions in compressive and tensile strength^20^. To overcome this limitation, the rubber fibers employed in this study were pretreated with a 1 M NaOH solution for 24 h, followed by thorough rinsing and air-drying. This alkali treatment has been shown to increase surface roughness, remove inert contaminants, and promote hydroxyl group formation, thereby improving adhesion between the rubber particles and the geopolymer gel^22^. When combined with fly ash-based geopolymer concrete, properly treated rubber fibers can provide crack-bridging effects, increase energy absorption, and delay microcrack coalescence under flexural loading without the severe strength penalties observed with untreated rubber. The rubber content in this study was intentionally limited to 9 kg/m^3^ to balance ductility enhancement against strength retention, consistent with previous studies indicating that low rubber replacement levels maintain matrix integrity while improving fracture behavior^24^.

This study aims to address the existing research gap by experimentally and theoretically evaluating the flexural behavior of precast geopolymer hollow-core slabs. The specific novel contributions of this work are: (1) First systematic comparison of solid OPC slabs versus solid GC slabs versus hollow-core GC slabs under identical loading and boundary conditions, enabling direct isolation of material and geometric effects; (2) Quantitative assessment of three void diameter levels (50 mm, 60 mm, 70 mm) on cracking load, ultimate capacity, effective stiffness, and crack width—providing design-relevant data that does not currently exist for GC hollow-core systems; (3) Experimental evaluation of three shear-span-to-depth ratios (a/d = 3.5, 5.0, and 7.0) for hollow-core GC slabs, elucidating the interaction between void geometry and span effects; (4) Validation that traditional flexural theory (ACI 318 and Eurocode 2) remains applicable to GC hollow-core slabs, which is non-trivial given the different tensile properties and elastic modulus of GC compared to OPC concrete.

By providing a detailed assessment of structural performance and crack characteristics, this study offers new experimental evidence supporting the feasibility of geopolymer concrete in precast hollow-core applications. The findings contribute to the advancement of sustainable structural systems and provide a valuable reference for the design of geopolymer hollow-core slabs in practical construction.

## Experimental study

### Materials

#### Cement

Ordinary Portland cement conforming to the specifications of ES 4756-1/2022^25^ was utilized to manufacture standard concrete of grade 35. The chemical makeup of all binder chemicals included in this study, including cement, FA, and SF, is detailed in Table 1.

#### Fly ash

It is FA type C in compliance with ASTM C618-19^26^, composed of light grey spherical particles, possessing a specific gravity of 2.0, with less than 10% retained on a 45-micron sieve. The chemical oxide composition of the employed fly ash (SiO_2_ + Al_2_O_3_ + Fe_2_O_3_) was 76.3%, with a loss on ignition (LOI) of 1.4%.

#### The alkali-activators

A 16 M NaOH solution was generated by dissolving 640 g of 97% pure NaOH pellets in deionized water^27^. The NaOH solution was made one day before its combination with Na_2_SiO_3_ to facilitate cooling. NaOH flakes were dissolved in tap water and swirled for three minutes. The activating solution was heated overnight in an oven at 75 °C to ensure complete dissolution of the NaOH solution and SF powder. The solution was subsequently maintained at room temperature for 24 h prior to utilization.

#### Aggregate

Natural siliceous sand with a fineness modulus of 2.85 was utilized as a fine aggregate. Crushed dolomite served as coarse aggregate, according to ASTM C33-07^28^, exhibiting a specific gravity of 2.65 and a fineness modulus of 7.87. Dolomite particle dimensions vary from 5 to 20 mm.

#### Rubber

**Fig. 1:**
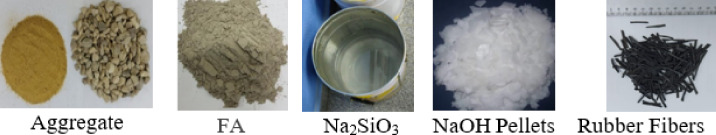
The used materials.

Rubber particles were incorporated in the geopolymer mix as a modification to improve ductility and energy absorption capacity. The rubber was sourced from used tires and subsequently processed manually by cutting it to the specified dimensions. The diameter and length of the rubber were 2 mm and 20 mm, respectively. The rubber fibers were submerged in a 1-molar NaOH solution for 24 h^22^. The rubber particles are thoroughly rinsed with regular water several times after 24 h of treatment until the pH of the wash water approaches neutrality. Subsequently, the processed rubber particles are air-dried at room temperature. Figure 1 illustrates the rubber fibers incorporated in the geopolymer mixtures, together with all components employed in the GC mix gradients.

#### Reinforcement

The reinforcement used was high grade steel with 10 mm diameter and yield strength of 400 MPa.

#### Plastic pipes

Plastic pipes of varying sizes (50 mm, 60 mm, and 70 mm) were utilized to form the longitudinal voids in the hollow-core slabs.

### Concrete mixes

The concrete grade utilized for the specimens is M 35. In GC mix the solid particles in the Na_2_SiO_3_ solution constitute 50.32% of the total solution, while water comprises 49.68%. Fine aggregate constitutes 35% of the overall aggregate. The overall water ratio is 0.25 of the binder weights^29^. The overall binder weight in Gc mix is 450 kg/m^3^, consisting of fly ash (FA). The alkaline ratio is 0.35 of the total binder weights, with sodium hydroxide (NaOH) constituting 50% of the alkaline component, amounting to 78.75 kg/m^3^.Table 1 presents the details of the GC and OPC mixes incorporating several variables.

**Table 1:** GC mix proportion (kg/m^3^) and Si/Al ratio.

Mix ID	FA	Cement	NaOH	Na_2_SiO_3_	AL ratio	Water in solution	Excess water	Sand	Coarse Agg.	Rubber	Si/Al ratio
OPC-mix	–	450	–	–	–	180	–	680	1,263	–	–
GC-mix	450	–	78.75	78.75	0.35	87.6	23.2	664	1,233	9.0	2.85

**Fig. 2:**
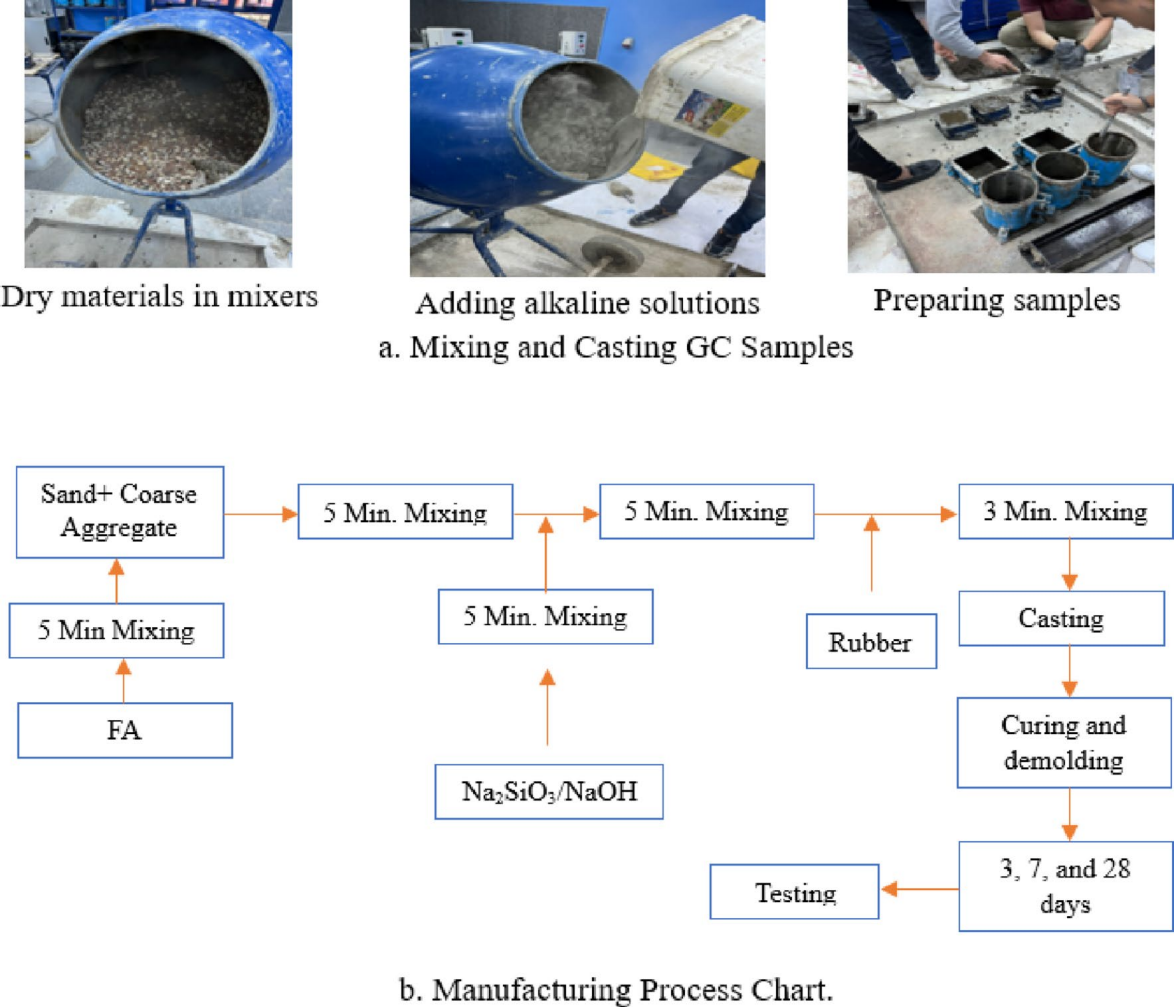
Laboratory manufacturingprocess for GC.

In GC mix, Aggregates were mixed in the drum mixer, then FA were added and mixed for five minutes, after that the activating solutions and the calculated excess water, which were gradually added and agitated for five minutes. finally, rubber was added to the mix for rubber GC mixes. To make concrete workable, superplasticizer is added to GC mixes, and the dosages was adjusted to achieve acceptable workability. The testing samples were cast. After then, all of the samples were vibrated for two minutes. Figure 2 shows the laboratory manufacturing process for GC mixes.

### Concrete and slab specimens

#### Concrete specimens

Cubic specimens (100 × 100 × 100 mm) were employed to ascertain the compressive strength in accordance with EN 12390 3:2002^30^. Cylinders (100 × 200 mm) were utilized to ascertain the splitting tensile strength in accordance with ASTM C 496-90^31^. Prisms of 100 mm x 100 mm x 500 mm were utilized to assess the flexural strength according to ASTM C 78-02^32^. Cylinders (150 × 300 mm) were utilized to ascertain the Young’s Modulus in accordance with ASTM 469-02^33^. All samples were maintained in the molds at ambient temperatures for 24 h prior to demolding. Following demolding, the control mix specimens (OPC) were immersed in water until the testing date, whereas the GC samples were exposed to ambient room temperature (25 °C) until testing. Three duplicates were conducted for each test, and the mean value was utilized throughout the study. The test results of concrete samples are presented in Table 2.

**Table 2:** Mechanical properties of concrete.

Mix type	PC slab samples		GC slab samples	
	7 days	28 days	7 days	28 days
Compressive strength (N/mm^2^)	26.4	35	33.28	37.17
Tensile strength (N/mm^2^)	3.8	4.9	4.99	6.06
Flexural strength (N/mm^2^)	3.9	5.5	6.16	7.40
Young’s Modulus (GPa)	-	26.80	-	25.88

The mechanical results unequivocally demonstrate that the geopolymer concrete (GC) mix surpasses the OPC mix in all strength classifications at both 7 and 28 days. This enhancement aligns with the GC mix proportions, wherein 450 kg/m^3^ of fly ash, in conjunction with NaOH and Na_2_SiO_3_ activators and a meticulously regulated alkaline liquid ratio, facilitates effective geopolymer gel formation. At 7 days, GC has markedly superior compressive, tensile, and flexural strengths relative to OPC concrete. The augmentation at an early age is attributed to the rapid dissolution of aluminosilicate phases and the generation of N-A-S-H / C-A-S-H hybrid gels, leading to a denser and more compact microstructure^]^. At 28 days, the GC samples surpass OPC performance, attaining compressive strength of 37.17 MPa, tensile strength of 6.06 MPa, and flexural strength of 7.40 MPa, hence affirming enhanced stiffness and superior fracture resistance. The significant enhancement in tensile and flexural strengths signifies improved binder-aggregate adhesion and less microcrack formation, aligning with the documented performance of optimally engineered low-calcium systems using fly ash geopolymer technology^35^. The lower rubber content (9 kg/m³) did not compromise strength; instead, it may improve ductility without compromising matrix integrity, in accordance with previous studies on low rubber replacement levels^24^.

The Young’s modulus of GC (25.88 GPa) is slightly lower than that of OPC (26.80 GPa), despite GC achieving higher strength values. This attitude aligns with prior data illustrating that GC generally attains 80–90% of the elastic modulus of OPC concrete with comparable compressive strength, attributable to variations in gel composition and microstructure. Olivia and Nikraz^36^ indicated that the electrical conductivity of 100% fly ash-based geopolymer concrete was 14.9–28.8% inferior to that of the ordinary Portland cement mix. GC exhibits a more ductile stress-strain response, consistent with the slight decrease in Ec observed in this work. The findings confirm that the selected GC mix proportions yield a structurally efficient binder with increased early strength, improved tensile capacity, and enhanced flexural performance compared to OPC concrete, aligning with documented trends in the literature.

#### Slab specimens

**Fig. 3:**
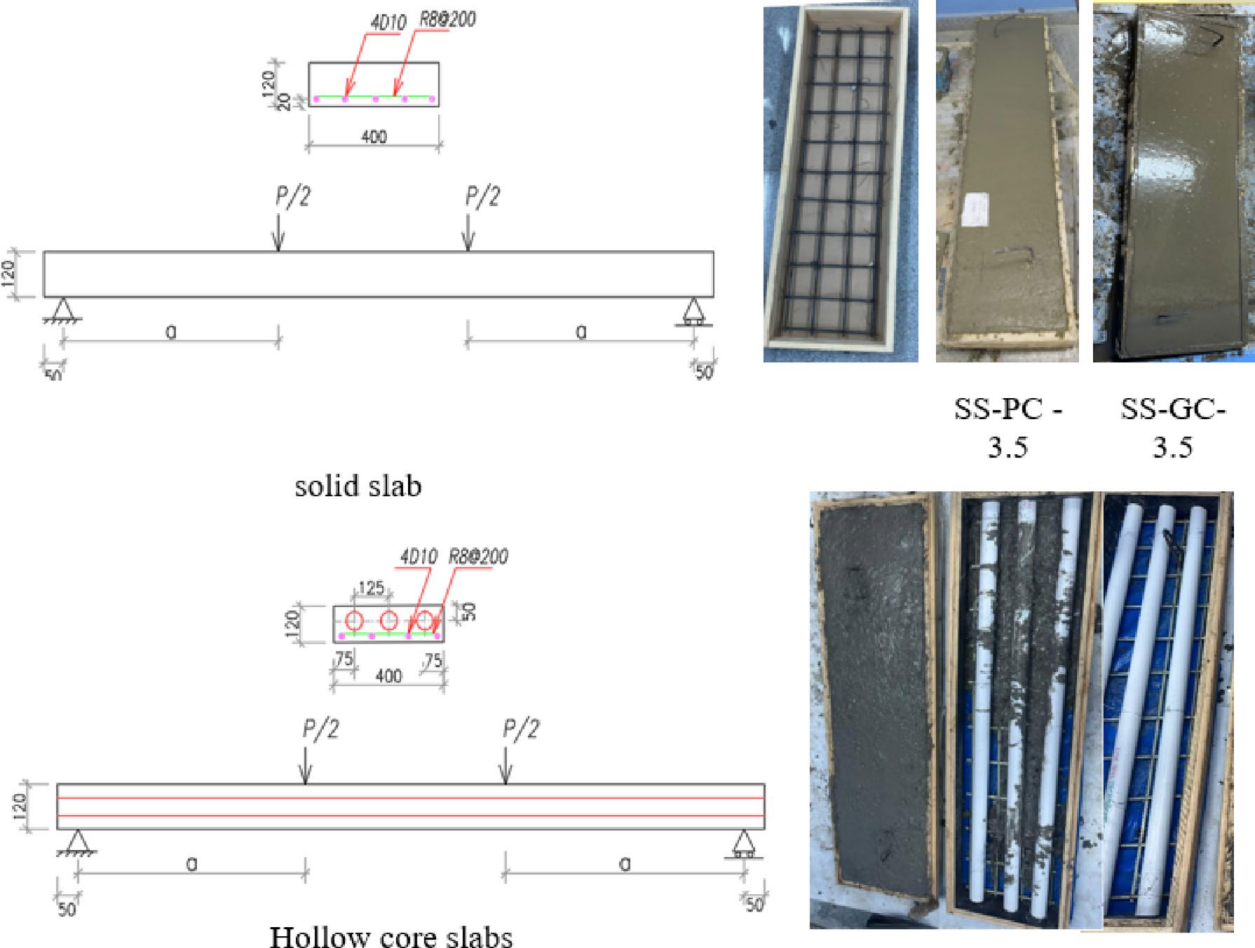
Casting, dimensions (mm) and details of reinforcement of slabs.

Seven reinforced concrete slabs were poured. The solid slabs, identified as SS-PC and SS-GC, were constructed from ordinary Portland cement (OPC) and geopolymer concrete (GC), respectively, measuring 120 × 400 × 1200 mm, with a fixed shear-span-to-depth ratio (a/d) of 3.5, equating to a shear span of 350 mm. The other specimens are hollow-core GC slabs, each measuring 120 × 400 × 1800 mm. Three specimens have void diameters of 50, 60, and 70 mm and are denoted as HC-D50, HC-D60, and HC-D70, respectively, and subjected to testing at an a/d ratio of 5. To further examine the impact of shear-span fluctuation, two additional HC-D50 specimens with a constant void diameter of 50 mm were evaluated at a/d ratios of 3.5 and 7.0. Table 3 delineates the specific characteristics of the seven slab specimens. Figure 3 depicts the casting, size (mm), and reinforcement details of the slabs.

**Table 3:** Details of the investigated slabs.

No.	Code	Slab type	Type	Reinf. (mm^2^)	a/d	a (mm)	Void Dim. (mm)	Total void ratio (%)	Slab Dim. (mm)
1	SS –PC-3.5	Solid	OPC	314	3.5	350	–	–	120 × 400 × 1200
2	SS –GC-3.5		GC	314	3.5	350			120 × 400 × 1200
3	HC-D50-5.0	Hollow	GC	314	5.0	500	D = 50	12.27	120 × 400 × 1800
4	HC-D60-5.0		GC	314	5.0	500	D = 60	17.66	120 × 400 × 1800
5	HC-D70-5.0		GC	314	5.0	500	D = 70	24.04	120 × 400 × 1800
6	HC-D50-3.5		GC	314	3.5	350	D = 50	12.27	120 × 400 × 1800
7	HC-D50-7.0		GC	314	7.0	700	D = 50	12.27	120 × 400 × 1800

#### Testing

**Fig. 4:**
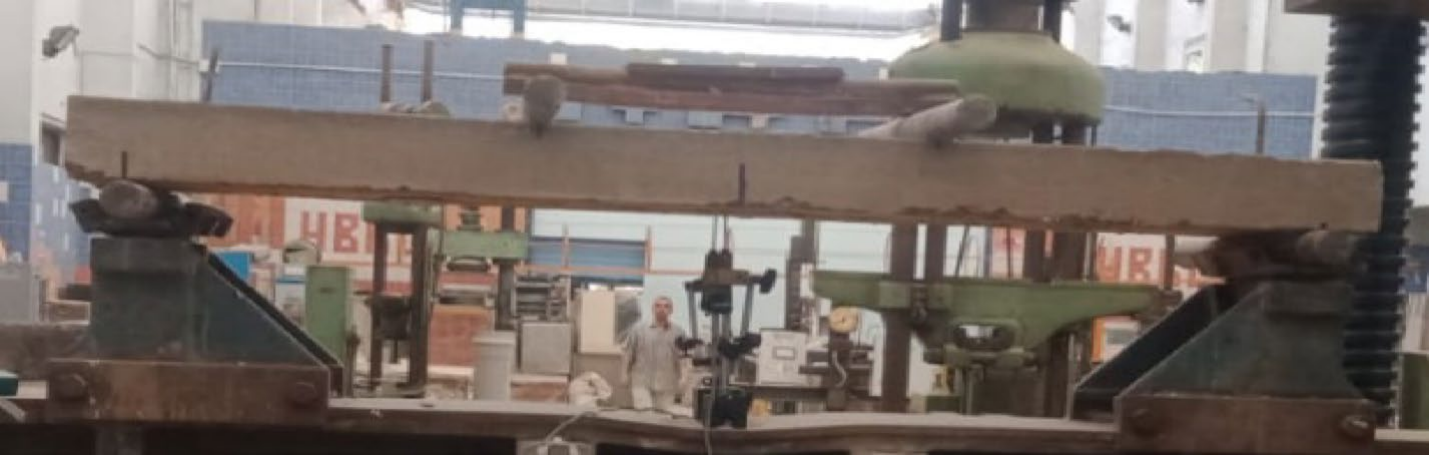
Loading scheme.

The test specimens underwent four-point loading across a simply supported span. The distance from the supports to the ends of the slabs is 50 mm at both extremities. The load was incrementally applied until failure occurred. During loading, observations and measurements encompassed crack patterns, central deflection, and failure causes. The deflection profile of the slabs was assessed using LVDTs positioned at mid-span, employing dial gauges with a sensitivity of 0.002 mm. The applied load, displacements, and strain measurements were electronically documented during the test via a data gathering system overseen by a computer. Figure 4 illustrates the loading process for test specimens.

## Experimental results and discussion

### Cracking behavior and ultimate loads

**Fig. 5:**
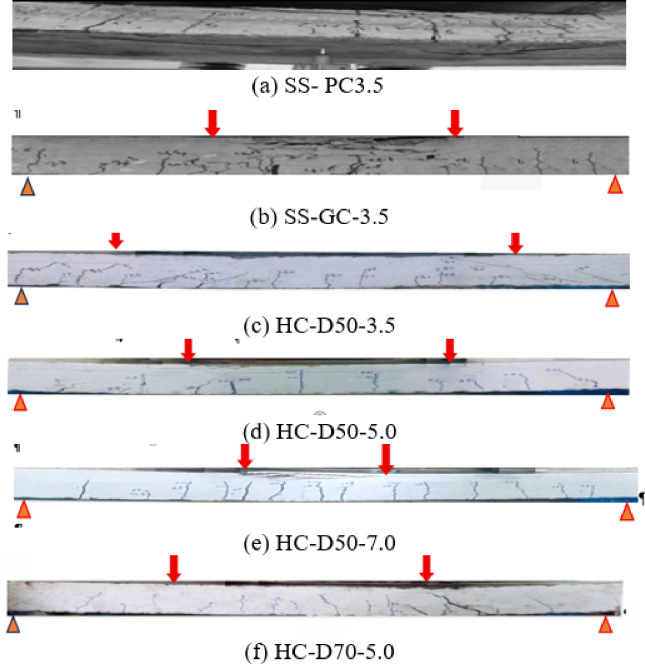
Crack pattern.

Figure 5 illustrates the crack pattern of the slabs, while Table 4 displays the estimated and experimental data for both cracking and ultimate loads. The determination of ultimate and cracking loads for the slabs was conducted utilizing reinforced concrete flexural design concepts in accordance with established regulations, including ACI 318^37^ and Eurocode 2^38^.

The ultimate moment capacity (M_u_) was calculated in Eq. (1), using the equilibrium of internal forces and the rectangular stress block method for concrete compression. The maximum compressive stress was set at 0.85f_c′_, which assumes a nonlinear stress distribution in concrete under ultimate load conditions. The formula used to determine the ultimate moment capacity is:

1 $${M_u} = {\rm{ }}{A_s} \times {\rm{ }}{f_y} \times {\rm{ }}\left( {d{\rm{ }} - \frac{a}{{2~}}} \right)$$

$$a=\:\:\frac{\mathrm{A}\mathrm{s}\times\:\mathrm{f}\mathrm{y}}{0.85\times\:\mathrm{f}\mathrm{c}{\prime\:}\times\:\mathrm{b}\:}$$

where A_s_ is the steel area f_y_ the steel yield strength, d is the effective depth of slab, f_c′_ the cylinder compressive strength of concrete, and *b* the effective width of the slab after void ratio adjustments for hollow core slabs.

The cracking moment M_cr_ was calculated in Eq. (2) based on the modulus of rupture (cracking tensile stress). The cracking stress was estimated as f_ctr_ = 0.62√f_c′_ where (f_c′_) is the compressive strength of concrete^37^. The cracking moment is given by:

2 $${{\rm{M}}_{{\rm{cr}}}} = \frac{{{\rm{fctr~Ig}}}}{{\rm{Y}}}$$

where (I_g_) is the gross moment of inertia of the uncracked section, and (Y) is the distance from the neutral axis to the extreme tensile fiber.

**Table 4:** Theoretical and experimental values for cracking and ultimate loads.

Slab code	Total Void ratio (%)	Effective width (mm)	Theoretical				Experimental	
			M_u_ (kN·m)	*P*_ult_ (kN)	M_cr_ (kN·m)	*P*_cr_ (kN)	*P*_ult_ (kN)	*P*_cr_ (kN)
SS–PC-3.5	-	400.00	10.90	62.30	3.78	21.60	58.80	14.80
SS–GC-3.5	-	400.00	11.00	62.86	3.89	22.26	60.00	16.20
HC-D50-5.0	12.27	351.00	11.00	44.00	3.71	14.83	41.80	9.90
HC-D60-5.0	17.66	329.00	11.00	44.00	3.51	14.03	40.80	9.60
HC-D70-5.0	24.04	304.00	11.00	44.00	3.18	12.71	39.20	8.30
HC-D50-3.5	12.27	351.00	11.00	62.86	3.83	21.90	58.10	13.00
HC-D50-7.0	12.27	351.00	11.00	31.43	3.83	10.95	30.00	7.00

In solid slab specimens, the bond between the steel reinforcement and the concrete was sufficient to guarantee continuous transmission of shear loads along the steel-concrete interface. Consequently, fracture spacing was predominantly governed by bond quality, with cracks originating at points where the shear stress transmitted from the longitudinal reinforcement exceeded the available bond strength. The shear-span-to-effective-depth ratio (a/d = 3.5) for the solid slabs approaches the shear-critical threshold, resulting in the observation of inclined shear fractures near the supports. Upon comparison of the solid slabs SS-PC and SS-GC depicted in Fig. 5a,b, it is clear that the SS-GC slab has finer cracks. This characteristic is due to the incorporation of rubber, which enhanced the stress-bridging capacity of the geopolymer matrix, facilitating scattered cracking instead of fewer, broader cracks. Figure 5a depicts the SS-PC slab, which has fewer but broader inclined cracks, whereas Fig. 5b represents the SS-GC slab, distinguished by numerous closely spaced cracks due to enhanced crack distribution characteristics. The augmented crack dispersion in the GC slab enhanced ductility and demonstrates that GC offers superior structural performance relative to OPC in solid slab applications.

The span-to-depth ratio (a/d) markedly affects the flexural performance of solid and hollow-core slabs. Slabs with elevated a/d ratios have diminished flexural stiffness, leading to augmented deflections and decreased cracking and ultimate loads. the comparison of HC-D50 slabs with a/d ratios of 3.5, 5.0, and 7.0 demonstrates that an increase in the a/d ratio from 3.5 to 7.0 results in a decrease in the experimental ultimate load from 58.1 to 30.0 kN and the cracking load from 13.0 to 7.0 kN. This reduction arises as a higher a/d ratio correlates with an extended effective span in relation to slab depth, diminishing the slab’s load capacity and facilitating premature cracking. The impact of a/d is more significant in hollow-core slabs with elevated void ratios, as the diminished cross-section exacerbates the reduction in flexural strength. Regulating the a/d ratio is essential in the design of hollow-core and geopolymer slabs to guarantee adequate flexural capacity and serviceability performance.

The overall void ratio, representing the proportion of the cross-section that is hollow, significantly influences both load-bearing capacity and cracking characteristics in hollow concrete slabs. As the void ratio escalates, for example, from 12.27% (HC-D50) to 24.04% (HC-D70), the effective concrete area diminishes, frequently becoming the slab more vulnerable to cracking and diminishing both shear and flexural capacities. Elevated void ratios lead to extended and more frequent crack formations, indicating diminished stress redistribution capacity and heightened susceptibility to brittle failure. This underscores the compromise between weight reduction and the necessity to maintain adequate concrete area for structural integrity in the design of hollow geopolymer concrete slabs. The current results align with previous experimental findings. Samer^3^ showed that circular voids preserved 88–90% of the strength of solid slabs, whereas square voids had greater decreases of 15–25%.

As indicated in Table 4, the solid slabs (SS–PC and SS–GC) exhibit a strong correlation between theoretical and experimental ultimate loads, with variations of less than about 7%. The GC solid slab (SS–GC-3.5) displays a somewhat superior experimental capacity (60.0 kN compared to 58.8 kN for SS–PC); this confirms the comparable or slightly enhanced mechanical performance of GC under flexural conditions.

In HCS, the theoretical cracking capacity (P_cr_) diminishes as the void diameter increases from D50 to D70, due to the reduction in the gross moment of inertia Ig. However, the theoretical ultimate capacity (P_ult_) remains identical across the HCS specimens. This is because the equivalent rectangular stress block depth (a), calculated per ACI 318-19, remains within the solid upper flange; thus, the theoretical ultimate flexural strength is not influenced by the void geometry in the tension zone.

While theoretical ultimate values remain constant, the experimental P_ult_ and P_cr_ values exhibit reductions of around 7–30% as the void ratio increases. This suggests that while the ACI simplified model treats the compression zone as a solid section, the physical increase in void ratio introduces experimental complexities, such as reduced net concrete area and stress concentrations that lead to the observed decrease in actual capacity.

The concordance between theoretical and experimental findings validates the analytical methodology. The geopolymer solid slab exhibits remarkable structural performance, and the behavior of hollow-core GC slabs is consistent with recognized mechanics. Specifically, the theoretical results remain stable across different void ratios because the compression zone is confined to the upper solid cord, as predicted by ACI 318 principles. This validates the appropriateness of GC for both solid and voided slab systems.

An increase in the total void ratio resulted in a consistent decrease in both cracking load and flexural capacity. Removing 12.27% (HC-D50-5.0), 17.66% (HC-D60-5.0), and 24.04% (HC-D70-5.0) of the concrete volume led to reductions in the experimental cracking load of 38.9%, 40.7%, and 48.8%, respectively, and decreases in ultimate strength of 30.3%, 32.0%, and 34.7% compared to the solid geopolymer control slab. This downward trend is attributed to the reduced moment of inertia caused by the longitudinal voids, which diminishes the flexural stiffness and net concrete area as the void diameter increases.

Solid slabs (SS–PC-3.5 and SS–GC-3.5) demonstrated an effective width of 400 mm, with experimental ultimate loads P_ult_ ranging from 58.80 to 60.00 kN and experimental cracking loads P_cr_ from 14.80 to 16.20 kN. The incorporation of longitudinal voids in hollow-core slabs (HC-D50, HC-D60, and HC-D70 series) reduced the effective width to between 351 mm and 304 mm, resulting in concomitant reductions in load capacity. For example, HC-D50-5.0 (12.27% void ratio) exhibited an experimental P_cr_ of 9.90 kN and a P_ult_ of 41.80 kN, representing a significant reduction compared to the solid control slabs. Subsequent increases in the void ratio to 17.66% (HC-D60-5.0) and 24.04% (HC-D70-5.0) resulted in further diminutions, yielding experimental cracking loads of 9.60 kN and 8.30 kN, and ultimate loads of 40.80 kN and 39.20 kN, respectively. These decreases are ascribed to the diminished moment of inertia and reduced net concrete cross-section associated with larger void sizes.

Geopolymer slabs (SS–GC-3.5) demonstrated marginally superior cracking and ultimate loads relative to conventional concrete (SS–PC-3.5). This suggests an enhanced tensile performance in the geopolymer binder, which helps mitigate the effects of the reduced cross-section in hollow-core designs. Notably, increasing the void diameter from 50 mm to 70 mm was found to affect the cracking load (P_cr_) more significantly than the ultimate load (P_ult_). The experimental results align closely with theoretical predictions, confirming that geopolymer hollow-core slabs maintain consistent structural performance even at higher void ratios. Additionally, variations in the span-to-depth ratio (a/d) in the HC-D50 series significantly influenced flexural behavior; specimen HC-D50-7.0 exhibited the lowest experimental ultimate load (30.0 kN) and cracking load (7.0 kN) due to the increased shear span, which reaches the section’s moment capacity at lower load levels.

The behavior of solid slabs with a/d ratio of 3.5 illustrated a close agreement between the conventional concrete (SS–PC-3.5) and geopolymer (SS–GC-3.5) slabs. The PC slab demonstrated a cracking load of 14.80 kN and an ultimate capacity of 58.80 kN, while the GC slab attained marginally superior values of 16.20 kN and 60.00 kN, respectively. Although both slabs were reinforced with 314.3 mm² of tensile steel and possessed identical geometric properties, the geopolymer mix which exhibited a slightly lower young’s modulus (25.88 GPa) but superior compressive strength (37.17 MPa) showed a slight improvement in both cracking and ultimate performance. These results suggest that geopolymer concrete can exhibit structural performance equivalent to, and in certain respects superior to, traditional Portland cement concrete. The findings provide additional evidence endorsing the feasibility of GC as a sustainable alternative for solid slab applications.

### Load-deflection relations

**Fig. 6:**
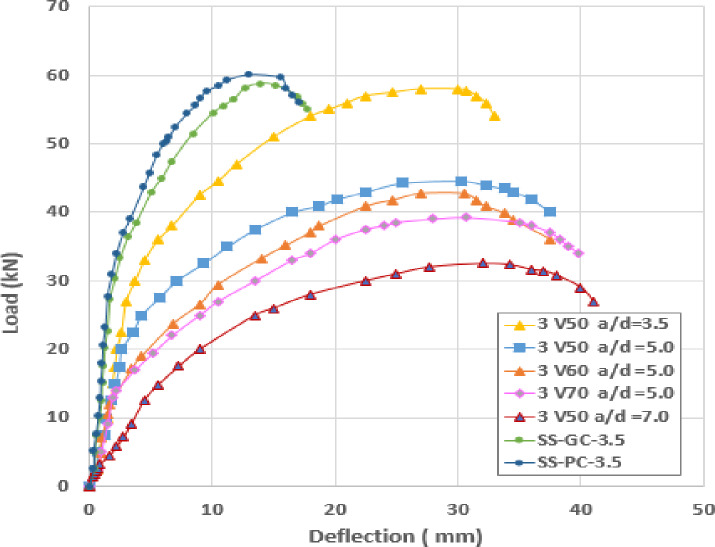
Load-deflection curve for slabs.

Figure 6 illustrates the load-deflection curves for the assessed slabs. Table 5 presents the theoretical and actual results for the ultimate displacement (δ _ult_) and the cracking displacement (δ_cr_). The slab deflection was calculated using classical elastic beam theory, as presented in Roark’s Formulas for Stress and Strain (Eq. 3)^39^, while the effective moment of inertia (I_e_) was determined according to ACI 318 provisions (Eq. 4).

3 $$\delta = \frac{{M}_{a}({3L}^{2}-{4a}_{v}^{2})}{\:24{\:E}_{c\:\:{I}_{e\:\:}\:}}$$

In the above equations δ is the mid-span deflection, L is the span length of the slab, and a_v_ represents the shear span (the distance from the support to the point of load application). The term “M_a_” denotes the applied bending moment, “E_c_” is the modulus of elasticity of concrete, and “I_e_” is the effective moment of inertia. The effective moment of inertia is evaluating using Eq. (4), Where I_cr_ is the cracked moment of inertia in the transformed section, and Ig is the gross (uncracked) moment of inertia. The term “M_cr_” denotes the cracking moment, which signifies the change from uncracked to cracked behavior. This formula guarantees that the calculated effective inertia is not above the gross inertia, thereby reflecting the stiffness reduction linked to flexural cracking.

4 $${{\rm{I}}_{\rm{e}}} = {\rm{ }}{\left( {{{\rm{M}}_{{\rm{cr}}}}/{{\rm{M}}_{\rm{a}}}} \right)^{\rm{3}}}{{\rm{I}}_{\rm{g}}} + \left[ {{\rm{1}} - {{\left( {{{\rm{M}}_{{\rm{cr}}}}/{{\rm{M}}_{\rm{a}}}} \right)}^{\rm{3}}}} \right]{\rm{ }}{{\rm{I}}_{{\rm{cr}}}}~~ \le {\rm{ }}{{\rm{I}}_{\rm{g}}}$$

$${I_{cr}} = \frac{{b{x^3}}}{3} + n{A_s}{(d - x)^2}$$

In the above equation, n is the modular ratio E_s_/E_c_ and can be approximately ≈ 10. The depth of the neutral axis (x) for the cracked section was determined using the transformed section method based on force equilibrium between concrete in compression and transformed steel in tension.

$$\frac{{b{x^2}}}{2} + n{A_s}(d - x)$$

**Table 5:** Predicted versus experimental deflection values.

Slab code	Total void ratio (%)	Theoretical					Experimental
		I_g_ (×10^7^ mm^4^)	I_cr_ (×10^7^ mm^4^)	I_eff_ (×10^7^ mm^4^)	δ_ult_ (mm)	δ_cr_ (mm)	δ_ult_ (mm)
SS–PC-3.5	–	5.76	1.50	3.46	7.75	2.25	13.00
SS–GC-3.5	–	5.76	1.55	3.52	7.81	2.36	13.92
HC-D50-5.0	12.27	5.48	1.10	2.90	24.36	5.63	30.30
HC-D60-5.0	17.66	5.18	0.90	2.53	29.72	5.95	30.50
HC-D70-5.0	24.04	4.69	0.70	2.04	38.78	6.57	31.00
HC-D50-3.5	12.27	5.67	1.55	3.46	18.96	5.77	30.00
HC-D50-7.0	12.27	5.67	1.55	3.46	15.94	4.85	34.25

The hollow-core slabs exhibited a clear sensitivity to both the shear span-to-depth ratio (a/d) and void diameter, as evidenced by their load and deformation characteristics. For slabs with a constant a/d ratio of 5.0, increasing the void diameter from 50 to 70 mm led to a gradual reduction in Pult from 41.80 kN (HC-D50-5.0) to 39.20 kN (HC-D70-5.0), accompanied by a minor increase in ultimate deflection from 30.3 mm to 31.0 mm. This pattern indicates that larger voids diminish the effective concrete area and stiffness, thereby reducing strength while allowing for slightly increased deformation. A comparable trend was observed for variations in the a/d ratio: the specimen with the lower ratio (HC-D50-3.5) achieved the highest ultimate load of 58.10 kN with a deflection of 30.0 mm, whereas the specimen with the highest ratio (HC-D50-7.0) exhibited a significantly diminished capacity of 30.00 kN and the greatest ultimate deflection at 34.25 mm. These results demonstrate that both elevated void ratios and increased $a/d$ ratios reduce load-carrying capacity while enhancing flexibility, suggesting that optimal void geometry and moderate shear-span ratios are crucial for preserving structural efficiency.

Interestingly, the theoretical models displayed a distinct inversion in prediction accuracy between the solid and hollow configurations. While the theoretical ultimate deflection for solid slabs was underestimated (approx. 7.81 mm), it was significantly overestimated for the hollow-core slabs, reaching 38.78 mm for specimen HC-D70-5.0 compared to the experimental 31.00 mm. This suggests that while the voids reduce the concrete cross-section, the PVC pipes used to form these voids provide an internal stiffening effect. Although this reinforcement is not sufficient to significantly impact the ultimate load-bearing capacity, it enhances the effective moment of inertia (I_eff_) and limits deformation, making traditional theoretical equations for I_eff_ notably conservative for these hollow-core designs.

### Crack width

Table 6 depicts the experimental (W_k Exp_) and anticipated (W_k Pred_.) values for crack width in PC and GC slabs. The evaluation of crack width in steel-reinforced concrete structures is important for ensuring the durability of structural elements, as it addresses the demands of structural integrity and serviceability standards. The maximum fracture spacing was determined using the Eurocode (EN 1992-1-1)^38^ formulation (Eqs. 5 and 6), which includes several empirical coefficients to consider bond properties and strain distribution. The coefficient k_1_ denotes the impact of bond characteristics between the reinforcement and concrete, with a standard value of 0.8 often assigned to high-bond (ribbed) steel bars. The parameter k_2_ addresses the uneven distribution of strain between concrete and steel; a value of 0.5 is advised for members primarily experiencing bending. The coefficient k_3_ denotes the influence of concrete cover on crack spacing, with a typical value of 3.4, signifying that greater cover distances generally augment the spacing between cracks. The value of coefficient k_4_, set at 0.425, signifies the cumulative effect of bar diameter and bond characteristics on crack development. The effective reinforcement ratio, ρ_s_,_eff_ is defined as the ratio of the area of tensile reinforcement to the effective concrete area in tension, influencing the interaction between steel and surrounding concrete; increased reinforcement ratios diminish crack spacing. The concrete cover c and bar diameter φ are crucial as they directly affect bond conditions and crack spacing. Collectively, these characteristics delineate the formula for maximum crack spacing and yield a dependable estimation of flexural crack widths in reinforced concrete components. While these coefficients are derived from conventional concrete standards, they provide a consistent baseline for evaluating the serviceability performance of geopolymer-based systems in this comparative study.

5 $${{\rm{S}}_{{\rm{r}},{\rm{ max}}}} = {{\rm{k}}_{\rm{3}}}{\rm{c}} + {{\rm{k}}_{\rm{1}}}{{\rm{k}}_{\rm{2}}}{{\rm{k}}_{\rm{4}}}\frac{{\rm{\varphi }}}{{{p_{s,eff}}}}$$

6 $${{\rm{w}}_{\rm{k}}} = ({\varepsilon _{{\rm{sm}} - }}{\varepsilon _{{\rm{cm}})}} \cdot {{\rm{s}}_{\rm{r}}}_{{\rm{max}}},~~~~~{\varepsilon _{\rm{s}}} = {\rm{ }}{\sigma _{\rm{s}}}/{\rm{ }}{{\rm{E}}_{\rm{s}}}$$

For the serviceability assessment, crack widths were evaluated at service stress levels (f_s_) of 120 MPa and 240 MPa. These values were selected to represent typical service load stages, with the 240 MPa level corresponding to approximately 65% of the reinforcement yield strength (f_y_ = 370 MPa). This ensures a clear distinction between the Serviceability Limit State (SLS) analysis and the Ultimate Limit State (ULS), where the full yield strength is utilized to determine the ultimate load-carrying capacity.

**Table 6:** Experimental and predicted values for crack width.

Slab Code	Effective width b (mm)	ρ_s_,_eff_	Theoretical			Exp. W_k_. (mm)
			s__r, max_ (mm)	w_k_ (mm) @ f_s_= 120 MPa	w_k_ (mm) @ fs= 240 MPa	
SS–PC-3.5	400	0.008	284.45	0.171	0.341	0.260
SS–GC-3.5	400	0.008	284.45	0.171	0.341	0.250
HC-D50-5.0	351	0.009	257.94	0.155	0.310	0.230
HC-D60-5.0	329	0.010	246.03	0.148	0.296	0.200
HC-D70-5.0	304	0.010	232.50	0.140	0.279	0.190
HC-D50-3.5	351	0.009	257.94	0.155	0.310	0.200
HC-D50-7.0	351	0.009	257.94	0.155	0.310	0.220

As demonstrated in Table 6, the theoretical and observed crack widths exhibit strong concordance across all specimens. For the solid slabs, SS–PC-3.5 and SS–GC-3.5 yielded theoretical widths of 0.171–0.341 mm, while experimental measurements were recorded at 0.26 mm and 0.25 mm, respectively. This slight variation suggests a potentially superior tensile performance in the geopolymer concrete matrix compared to the OPC control. The hollow-core slabs exhibited experimental widths that were consistently lower than the theoretical upper-bound projections; for example, HC-D50-5.0 showed an experimental value of 0.23 mm (versus 0.31 mm theoretical), and HC-D70-5.0 recorded 0.19 mm (versus 0.279 mm theoretical). These results indicate that an increase in void diameter, which elevates the effective reinforcement ratio (ρ_s_,_eff_), may moderately mitigate crack width development.

The discrepancies between experimental and theoretical values were confined to a narrow range of 0.05–0.08 mm, affirming the dependability of the Eurocode-based methodology for these applications. This illustrates that the adopted formulae for maximum crack spacing and crack width are suitable for the serviceability evaluation of both solid and hollow-core geopolymer slabs. Furthermore, the inclusion of a minor rubber content in the GC mix appears to yield modest enhancements in post-cracking deformability without an associated increase in crack width. This behavior could be attributed to improved energy absorption and the postponement of microcrack propagation by the rubber particles, though further microstructural characterization is required to confirm this mechanism. Overall, the findings validate that the proposed formulas are appropriate for practical serviceability assessments of sustainable solid and hollow-core slabs.

The experimental observations, such as the refined crack patterns and the consistent load-deflection response of the geopolymer slabs, suggest potential enhancements in bond behavior and stress distribution within the geopolymer matrix. However, it is important to note that the current experimental program focused on macro-structural performance. In the absence of direct reinforcement strain measurements or microstructural interface analyses, these observations are presented as likely contributing factors rather than established mechanisms. The performance of the geopolymer-steel interface remains a subject for detailed investigation.

## Conclusions

Based on the experimental and theoretical investigation of geopolymer concrete solid and hollow-core slabs, the following key conclusions are drawn:

1. GC vs. OPC performance: The geopolymer concrete solid slab (SS-GC-3.5) exhibited marginally superior flexural performance compared to its OPC counterpart, achieving a 9% higher cracking load (16.2 kN vs. 14.8 kN) and 2% higher ultimate load (60.0 kN vs. 58.8 kN). This confirms that properly formulated GC is a structurally equivalent—and in some respects superior—alternative to OPC concrete for slab applications.
2. Effect of void ratio: Increasing the total void ratio from 12.27% (D50) to 24.04% (D70) in hollow-core slabs reduced the experimental cracking load by 14% (from 14.83 kN to 12.71 kN) and the ultimate load by 6% (from 41.8 kN to 39.2 kN). These reductions are attributed to diminished effective moment of inertia and flexural stiffness.
3. Effect of shear-span ratio: The shear-span-to-depth ratio (a/d) dramatically influences capacity. For HC-D50 slabs, increasing a/d from 3.5 to 7.0 reduced the ultimate load by 48% (from 58.1 kN to 30.0 kN). Designers of hollow-core GC slabs must therefore carefully control span-to-depth relationships to avoid premature flexural failure.
4. Analytical validation: Theoretical predictions using traditional flexural theory (ACI 318 and Eurocode 2) showed strong agreement with experimental results for both solid and hollow-core GC slabs, with discrepancies typically under 10%. This validates that existing analytical framework remain applicable to geopolymer-based voided slab systems.
5. Serviceability and sustainability: Maximum crack widths remained below 0.35 mm for solid GC slabs and 0.31 mm for hollow-core GC slabs under service loads, demonstrating acceptable serviceability performance. The use of GC reduces cement consumption and utilizes industrial by-products (fly ash, waste rubber tires), offering a structurally efficient and environmentally sustainable solution for precast floor systems.

6- It should be noted that the geopolymer concrete mix used in this study includes rubber particles, which may contribute to the overall structural response. Therefore, the observed behavior reflects the combined effect of the geopolymer binder system and rubber modification.

## Recommendations for further study

Based on the findings and limitations of this study, the following areas are recommended for further research:

1. Bond-slip characterization: Specific bond-strength testing is required to quantify the interface behavior between the geopolymer matrix and steel reinforcement.
2. Strain analysis: Future studies should incorporate reinforcement strain gauges to map the internal stress distribution and verify ductility enhancement mechanisms.
3. Microstructural analysis: Investigation of the Interfacial Transition Zone (ITZ) and fracture surface characterization would help substantiate the stress-bridging effects observed in these slabs.
4. Void-former contribution: Further research is needed to isolate and quantify the stiffening effect provided by permanent PVC void-formers on the serviceability limits of hollow-core systems.”

## Data Availability

All data generated or analyzed during this study are included in this published article.
